# Prenatal and adult exposure to smoking and incidence of type 1 diabetes in children and adults–a nationwide cohort study with a family-based design

**DOI:** 10.1016/j.lanepe.2023.100775

**Published:** 2023-11-04

**Authors:** Yuxia Wei, Jessica Edstorp, Maria Feychting, Tomas Andersson, Sofia Carlsson

**Affiliations:** a Institute of Environmental Medicine, Karolinska Institutet, Box 210, Stockholm S-171 77, Sweden; b Center for Occupational and Environmental Medicine, Region Stockholm, Solnavägen 4, Stockholm S-113 65, Sweden

**Keywords:** Type 1 diabetes, Smoking, Incidence, Epidemiology

## Abstract

**Background:**

Prenatal exposure to smoking is linked to a reduced risk of type 1 diabetes in children. We wanted to find out if the risk of adult-onset type 1 diabetes is reduced in individuals who are exposed to smoking prenatally or during adulthood.

**Methods:**

We linked Swedish, nationwide registers and prospectively analyzed incidence of type 1 diabetes in relation to maternal smoking during pregnancy and adult smoking. Everyone was followed until age 30 or year 2019. We employed cohort and sibling design and used adjusted Cox regression and conditional logistic regression.

**Findings:**

For analyses of maternal smoking there were 3,170,386 individuals (18,745 cases of type 1 diabetes) and for adult smoking 1,608,291 individuals (1274 cases). Prenatal exposure to smoking was associated with lower incidence of type 1 diabetes during childhood and young adulthood (age 20–24, Hazard ratio (HR) 0.76, 95% Confidence interval 0.67–0.87), but not at higher ages. The HR associated with adult smoking was estimated at 1.14 (CI 1.00–1.31) overall and 1.34 (CI 1.03–1.75) in those with family history of diabetes. In sibling analyses, the odds ratio (OR) of type 1 diabetes in relation to prenatal exposure was 0.71 (CI 0.62–0.81) in children and 1.06 (CI 0.75–1.51) in adults (age 19–30), while adult smoking conferred an OR of 1.59 (CI 1.08–2.35).

**Interpretation:**

These findings indicate that a reduced risk conferred by tobacco exposure is limited to the prenatal period and type 1 diabetes developing during childhood. Adult smoking may be a risk factor for adult-onset type 1 diabetes, especially in people with family history of diabetes.

**Funding:**

Swedish Research Councils, Swedish Diabetes and Novo Nordisk Foundations, 10.13039/501100004543China Scholarship Council.

**Research in context**

**Evidence before this study**

Prenatal exposure to smoking and incidence of type 1 diabetes in children has been investigated in 23 observational studies according to a systematic review and meta-analysis published in 2022. No previous study has investigated incidence of adult-onset type 1 diabetes in relation to prenatal exposure to smoking. To identify studies investigating adult smoking exposure in relation to incidence of adult-onset type 1 diabetes, a literature search was made in PubMed in February of 2023 using the search terms (smoking) AND (incidence) AND (type 1 diabetes) AND (adults) AND (risk). The search retrieved 565 papers and only one of them, based on 32 cases, provided data on the association between smoking and incidence of type 1 diabetes.

**Added value of this study**

This study shows that if smoking has the capacity to reduce the risk of type 1 diabetes, this only relates to prenatal exposure and type 1 diabetes with onset during childhood, not adulthood. It is the first study to suggest that smoking increases the risk of adult-onset type 1 diabetes. By combining traditional cohort analyses with a sibling design, which can account for environmental and genetic factors shared between siblings, we can minimize potential confounding, and increase the evidentiary value of these findings.

**Implications of all the available evidence**

A reduced risk of type 1 diabetes in children exposed to maternal smoking does not have any immediate clinical implications considering the range of severe, adverse health effects attributable to such exposure. However, our findings point at the prenatal period as a crucial stage in disease development, and a need for further studies to elucidate if nicotine could have beneficial effects on development of islet autoimmunity. We also find support for smoking as a modifiable risk factor for adult-onset type 1 diabetes, contributing to the limited understanding of the etiology of type 1 diabetes in adults.

## Introduction

Prenatal exposure to smoking is linked to a reduced risk of type 1 diabetes in children. A meta-analysis including prospective studies from Europe, Australia, and the US reports 22% lower risk in children carried by smoking compared to non-smoking mothers.^1^^,^^2^ These findings were replicated in a quasi-experimental study that compared children who were prenatally exposed to smoking to their non-exposed siblings, thereby accounting for the influence of shared genetic and environmental factors.^3^ Early life factors are shown to influence the risk of developing chronic diseases across the life course such as low birth weight which is inversely related to type 2 diabetes that develops past middle age.^4^ Thus, it seems possible that prenatal smoking exposure could have long-lasting benefits that also reduce the risk of acquiring type 1 diabetes during adulthood. This, however, has never been investigated. Whether the influence of maternal smoking on offspring diabetes risk is modified by heritability to diabetes also remains to be explored.

The mechanism linking maternal smoking to type 1 diabetes in the offspring is unknown, but it has been proposed that a protective effect could reflect immunosuppressive properties of nicotine.^5^^,^^6^ If prenatal exposure to smoking prevents or delays onset of type 1 diabetes this may also relate to smoking during adulthood. Consequently, the risk of adult-onset type 1 diabetes may be reduced in smokers. A Norwegian study observed a reduced risk of type 1 diabetes in heavy smokers, but it was based on only 32 incident cases.^7^ We are not aware of any other study on the topic.

To fill this knowledge gap, we set out to investigate prenatal exposure to smoking and adult smoking behavior in relation to the incidence of type 1 diabetes in children (only prenatal smoking) and adults. We also investigated potential effect modification by family history of diabetes. To this aim, we used nationwide data from Swedish national registers and employed family-based designs in addition to regular cohort designs to control for familial confounding.

## Material and methods

### Study population

**Fig. 1:**
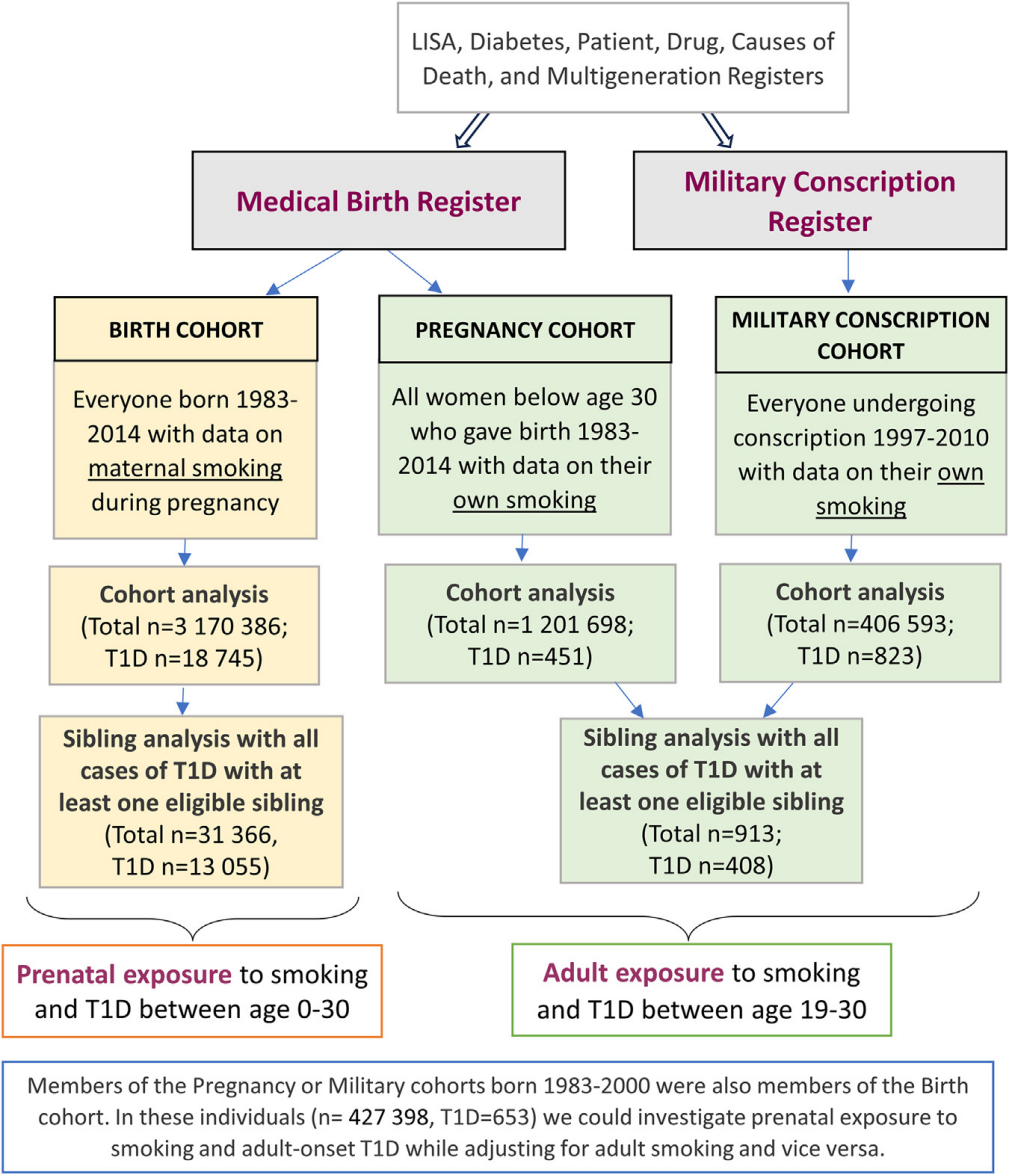
Study design.

We linked data from the Swedish National Population, Prescription, Patient, Cause of Death, Diabetes, Multigeneration, Medical Birth and Military Conscription Registers and the Longitudinal integration database for health insurance and labor market studies (LISA) (Fig. 1). The linkage was achieved through the unique personal identification number assigned to every Swedish citizen. To study prenatal exposure to smoking we used a study population consisting of all individuals recorded in Medical Birth Register who were born 1983–2014 (n = 3,170,386), referred to as the “Birth Cohort”. Two study populations were used for analyses of adult smoking; the first called the “Military Conscription Cohort” consisted of all individuals who completed military conscription between 1997 and 2010 with recorded information on smoking (n = 406,593, 97.1% men). To get corresponding information for women, we created a “Pregnancy Cohort” including all women who gave birth before age 30, between 1983 and 2014 with information on smoking recorded in the Medical Birth Register (n = 1,201,698). Information on both prenatal and adult exposure to smoking was available for 427,398 (26.6%) individuals in the Pregnancy and Military Conscription Cohorts who were born 1983 onwards and therefor had information on their mother recorded in the Medical Birth Register.

### Information on smoking and co-variates

The Medical Birth register records standardized information from prenatal exams for all women who give birth in Sweden. Information on smoking has been collected since 1983 and includes smoking status three months prior to pregnancy (since 1999), at enrollment which typically is at 8–12 weeks of pregnancy (since 1983) and gestational week 30–32 (since 1990). The information is recorded as non-smoking, smoking 1–9, or ≥10 cigarettes per day. This register provided information on prenatal exposure to smoking in individuals born since 1983 and information on adult smoking in women who gave birth from 1983 onwards. The validity of self-reported smoking information in the Medical Birth Register is high according to a study that compared self-reports to cotinine measurements.^8^ Maternal smoking was defined as smoking at the first prenatal visits and adult smoking as reporting smoking at any visit. If a woman had information from several births, we used the first recording in the analyses of adult smoking. Height and weight are recorded in the first trimester and used to calculate body mass index (BMI).

The Military Conscription Register holds information from the conscription examination that was mandatory for all Swedish men until July 1, 2010.^9^ The majority attended at age 18 and it was completed by > 95% of Swedish males. Self-reported smoking is recorded since 1997 and classified as none, 1–10, 11–20, >20 cigarettes/day. There is also information of use of Swedish smokeless tobacco “snus” classified as none, 1, 1–2, >2 packages per week. Information on BMI is available based on recordings of height and weight, and physical fitness, assessed through maximal aerobic workload (W_max_) obtained through cycle ergometer testing and muscle strength assessed through the ISOKAI isokinetic lift test.^9^

Information on education for the members of the study populations and their parents was obtained by linkage to LISA. LISA integrates data from the educational and labor market and holds annual registers since 1990 for all Swedish citizens, ≥16 years of age.

### Information on diabetes

The study populations were followed for a diagnosis of type 1 diabetes until age 30 in the Patient, Diabetes and Prescribed Drug Registers. The Patient Register holds information on all diagnoses, coded according to the Swedish version of the International Classification of Disease (ICD-10 since 1997), from hospital admissions since 1987 and outpatient specialist care since 2001.^10^ The Diabetes Register started in 1996 and collects information on diabetes care from Swedish health centers, including characteristics of the patients.^11^ It covers 90% of all patients. The Prescribed Drug Register records all filled prescriptions since July 2005, based on the Anatomical Therapeutic Chemical (ATC) classification system.^12^ We identified incident cases of type 1 diabetes based on information from the Diabetes (record of type 1 diabetes) and Patient Registers (ICD-8/ and ICD-9 code 250, or ICD-10 code E10). When a case was initially diagnosed using ICD-8/ICD-9, a later record of ICD-10 code E10 was used as a confirmation of type 1 diabetes. The date of diagnosis was defined as the first recording in the Diabetes, Patient or Prescribed Drug Register. Individuals with a record of type 2 diabetes (ICD-10 code E11) or unknown type of diabetes at any time, in either register during follow-up, were censored. All individuals with a recorded diagnosis of diabetes or a record of glucose lowering drugs before baseline (ATC code A10) were excluded. If a parent had a diagnosis of diabetes or a recording of glucose lowering medication at any time in the Patient, Diabetes or Prescribed Drug Register, the subject was classified as having family history of diabetes.

### Family-based, nested case–control study

We used the Multigeneration Register, which contains information on parents of all individuals born since 1932, to link siblings within the Birth, Pregnancy and Military Conscription Cohorts (Fig. 1). In the sibling comparison design, we compared individuals with type 1 diabetes to their siblings (same mother) who were alive and free of diabetes at the age when the case was diagnosed. The association of prenatal or adult exposure to smoking with type 1 diabetes was estimated by comparing siblings discordant both on exposure and outcome. However, siblings that were concordant on the exposure were informative for the estimates for covariates and were therefore included as participants. This type of analysis inherently adjusts for unmeasured confounders that are shared between siblings, including both genetic and environmental factors. When the estimate in the sibling comparison design approaches 1, it indicates a greater probability that the association detected in the overall cohort analysis is attributed to confounding factors shared among siblings.^13^

### Statistical analysis

#### Cohort analyses

In the analysis of smoking vs. non-smoking and incidence of type 1 diabetes, person time was generated from either birth (Birth Cohort) or baseline (Military Conscription and Pregnancy Cohort), until age at diabetes diagnosis, death, migration, age 30, or December 31st, 2019, whichever came first. Discrete-time proportional hazards regression models estimated hazard ratios (HRs) and 95% confidence intervals (CIs) of type 1 diabetes in relation to prenatal or adult exposure to smoking and snus use (only Military Conscription Cohort) with age as the underlying time scale. The main model (Model 2) was adjusted for sex, calendar year (continuous), family history of diabetes (yes vs. no), maternal BMI (for the exposure of maternal smoking) or adult BMI (<25, 25–29.9 and ≥30; for the exposure of adult smoking) and highest parental education (primary school, secondary school, or university). Based on the same regression model (Model 2), we estimated the incidence of type 1 diabetes from birth to age 30 (per month) separately in those exposed vs. unexposed to maternal smoking. The analyses of the Military Conscription Cohort were additionally adjusted for physical fitness (continuous), muscle strength (continuous) and snus use (yes vs. no). Participants with missing values on covariates were treated as a separate group in the analyses. Adult smoking was analyzed separately in the Military Conscription and Pregnancy Cohort, as well as in the two cohorts combined. In those analyses, women who appeared in both cohorts were removed from the Pregnancy Cohort. We analyzed prenatal exposure to smoking in relation to type 1 diabetes diagnosed at different ages. We also performed all analyses (model 2) in those with and without a family history of diabetes separately.

#### Sibling comparison analyses

In the sibling analyses, prenatal exposure to smoking was analyzed in the Birth Cohort and adult exposure to smoking was analyzed in the Pregnancy and Military Conscription Cohorts combined. We used conditional logistic models to estimate odds ratios (ORs) and 95% CIs of type 1 diabetes in relation to maternal and adult smoking while conditioning on sibling groups. The siblings were matched on age and the ORs were adjusted for sex. Analyses of adult smoking were also adjusted for BMI.

#### Sensitivity analyses

We investigated the association between prenatal exposure to smoking and adult-onset type 1 diabetes while adjusting for own smoking and BMI in the subset with information on both covariates (described in the study population section). In this subset we could also investigate incidence of adult-onset type 1 diabetes in relation to the combination of maternal and adult smoking status. In the Military Conscription Cohort, we could investigate the association between type 1 diabetes incidence and the combination of adult snus use and smoking. We performed complete cases analyses to ensure that the results were not biased by missingness on covariates. To check for residual confounding, we performed sensitivity analyses where we included early life factors (maternal age at delivery, birth order, gestational age and birth weight for gestational age) in model 2 together with maternal/own country of birth. We used a stricter definition of type 1 diabetes and excluded anyone who had ever been prescribed an oral glucose lowering drug (ATC code A10B) from 2005 (start of the Prescribed Drug Register) onwards.

### Role of the funding source

The funders did not have any influence on any aspect of the work.

## Results

### Characteristics

Prenatal exposure to smoking was investigated in the Birth Cohort where 18,745 (0.6%) individuals developed type 1 diabetes before age 31 of whom 2514 (16.3%) were diagnosed between ages 19 and 30 (Appendix Table S1). Among those who developed diabetes, a larger proportion had a parent with diabetes than in the total Birth Cohort, whereas no clear differences were seen regarding parental educational level.

We investigated adult smoking in 1,608,291 individuals (Pregnancy Cohort: n = 1,201,698, Military Cohort: n = 406,593) followed between age 19 and 30, during which 1274 (0.08%) (Pregnancy Cohort: n = 451 and Military Cohort: n = 823) developed type 1 diabetes. The Military Conscription Cohort was younger at baseline (mean age 18.3 vs. 25.7 years) and had lower prevalence of obesity and family history of diabetes compared to the Pregnancy Cohort. In the Military Conscription Cohort, 14.9% were smokers and 25.3% used snus, while in the Pregnancy Cohort 25.1% were smokers. People who developed type 1 diabetes were more likely to have parents with diabetes and in the Pregnancy Cohort, they less frequently had high education and were more likely to be obese and smoke at baseline.

The sibling analyses of prenatal exposure to smoking included 13,055 individuals with type 1 diabetes with at least one eligible sibling (n = 18,311) and those of adult smoking included 408 individuals with type 1 diabetes with at least one eligible sibling (n = 505) (Appendix Table S2).

### Prenatal exposure to smoking and type 1 diabetes

**Fig. 2:**
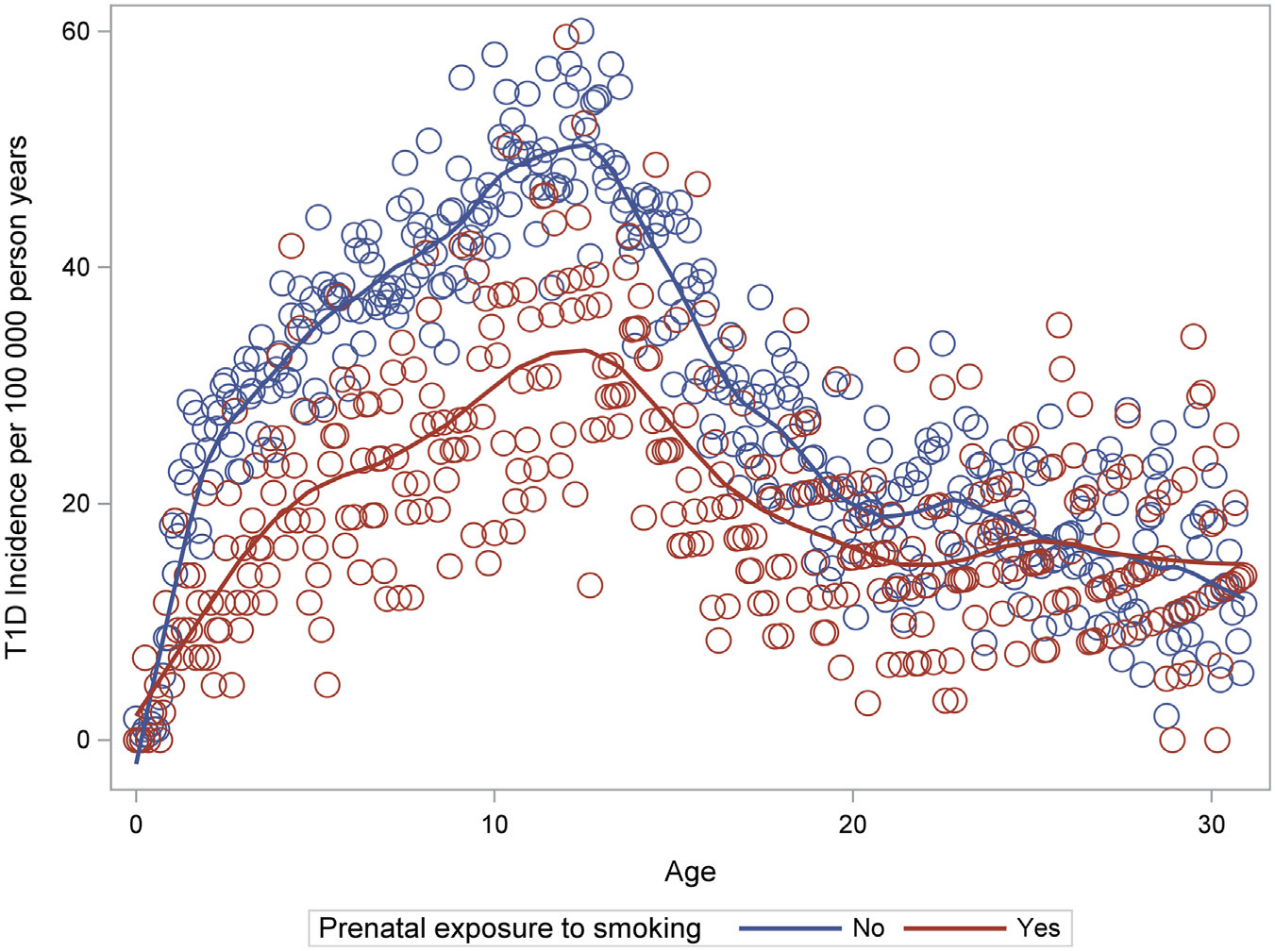
Incidence of type 1 diabetes (per 100,000 person years) by age and prenatal exposure to smoking (yes vs. no). The dots represent incidence estimates by age in months together with smoothed curves (LOESS-locally estimated scatterplot smoothing).

**Fig. 3:**
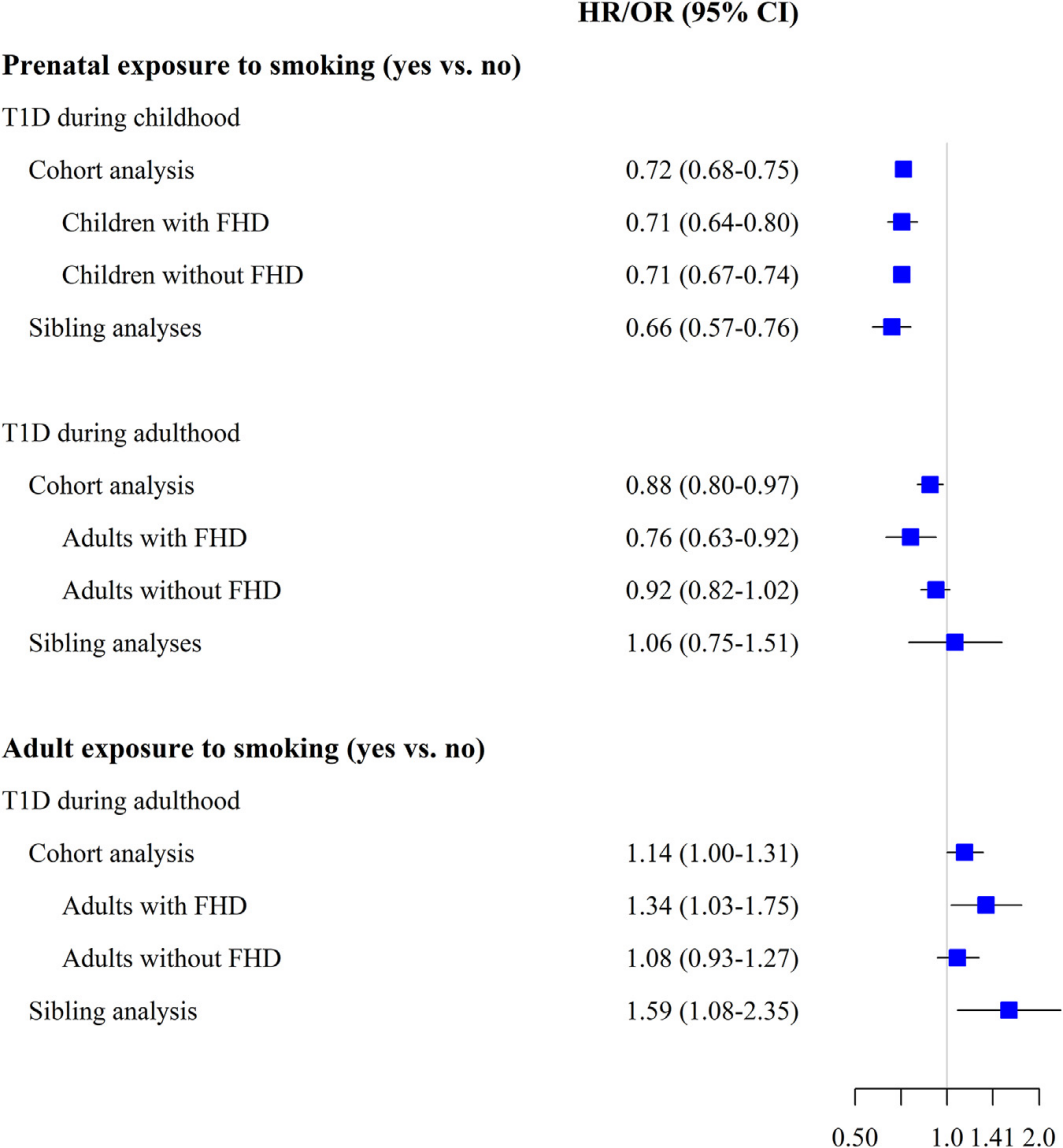
Prenatal and adult exposure to smoking and incidence of type 1 diabetes during childhood and adulthood. Cohort analysis, overall and by family history of diabetes together with sibling analyses. The analyses of prenatal exposure were based on the Birth Cohort and those of adult exposure on combined data from the Military Conscription and Pregnancy Cohorts. Cohort analyses were adjusted for age (time axis), calendar year, sex, parents' education, maternal BMI, or adult BMI. CI (Confidence interval), FHD (Family history of diabetes), HR (Hazard ratio), OR (Odds ratio), T1D (type 1 diabetes).

Incidence increased with age up to a peak at 10–14 years (Fig. 2). This pattern was seen both in children exposed and unexposed to maternal smoking, although incidence appeared to be higher in the unexposed children throughout childhood. Maternal smoking vs. non-smoking during pregnancy was associated with a lower incidence of type 1 diabetes between age 0 to 18 (HR 0.72, CI 0.68–0.75) and between age 19–30 (HR 0.88, CI 0.80–0.97) in the cohort analyses (Table 1, Fig. 2). The latter results were unaffected by adjustment for participants’ own smoking and BMI (Appendix Table S3). Sibling analyses yielded similar results for maternal smoking and type 1 diabetes in children (OR 0.71, CI 0.62–0.81), but no reduced risk of adult-onset type 1 diabetes was seen (OR 1.06, CI 0.75–1.51). Performing the cohort analysis by finer age groups revealed a 24–30% lower incidence of type 1 diabetes in the offspring of smoking compared to non-smoking mothers until age 24, whereas no difference was observed between age 25–30 (HR 0.96 CI, 0.83–1.12) (Table 1). Corresponding sibling analysis, comparing those exposed to maternal smoking to their unexposed siblings, revealed a reduced risk until age 10–14 (OR 0.55, CI 0.43–0.72), a tendency toward a reduced risk between age 15 and 19, but no risk reduction was seen at higher ages (e.g., OR was 1.01, CI 0.64–1.62 between age 20–24) (Table 1). Prenatal exposure to smoking was associated with a reduced risk of childhood type 1 diabetes in people with as well as without family history of diabetes (Fig. 3).

**Table 1:** Prenatal exposure to smoking and incidence of type 1 diabetes between ages 0 and 30: Cohort and sibling analyses.

	Cohort analyses				Sibling analyses		
	Person-years	No. cases	HR (95% CI) Model 1	HR (95% CI) Model 2	No. siblings	No. cases	OR (95% CI)
**Type 1 diabetes between age 0 and 30**							
Maternal non-smoking	51,523,059	16,117	ref	ref	16,029	11,572	ref
Maternal smoking	12,766,510	2628	0.76 (0.73–0.79)	0.74 (0.71–0.77)	2282	1483	0.71 (0.62–0.81)
1–9 cigarettes/day	8,044,507	1727	0.78 (0.74–0.82)	0.77 (0.73–0.81)	1461	998	0.73 (0.64–0.84)
≥10 cigarettes/day	4,722,003	901	0.72 (0.67–0.77)	0.69 (0.65–0.74)	821	485	0.60 (0.50–0.74)
**Type 1 diabetes between age 0 and 18**							
Maternal non-smoking	40,691,123	14,179	ref	ref	14,305	10,309	ref
Maternal smoking	9,063,279	2052	0.73 (0.70–0.76)	0.72 (0.68–0.75)	1894	1181	0.66 (0.57–0.76)
1–9 cigarettes/day	5,761,034	1380	0.76 (0.72–0.81)	0.75 (0.71–0.80)	1227	806	0.69 (0.59–0.80)
≥10 cigarettes/day	3,302,245	672	0.67 (0.62–0.72)	0.65 (0.60–0.70)	667	375	0.55 (0.44–0.68)
**Type 1 diabetes between age 19 and 30**							
Maternal non-smoking	10,831,936	1938	ref	ref	1724	1263	ref
Maternal smoking	3,703,231	576	0.90 (0.82–0.99)	0.88 (0.80–0.97)	388	302	1.06 (0.75–1.51)
1–9 cigarettes/day	2,283,473	347	0.88 (0.78–0.98)	0.86 (0.77–0.97)	234	192	1.08 (0.75–1.54)
≥10 cigarettes/day	1,419,758	229	0.94 (0.82–1.07)	0.90 (0.79–1.04)	154	110	1.00 (0.62–1.62)
**Maternal smoking vs. non-smoking and type 1 diabetes by age**							
Age 0–4 years	15,794,419	3199	0.74 (0.67–0.83)	0.73 (0.66–0.82)	3432	2438	0.89 (0.64–1.26)
Age 5–9 years	14,437,819	5088	0.72 (0.66–0.78)	0.71 (0.65–0.77)	5466	3923	0.58 (0.45–0.76)
Age 10–14 years	11,792,969	5000	0.74 (0.68–0.80)	0.72 (0.66–0.78)	5226	3668	0.55 (0.43–0.72)
Age 15–19 years	9,440,519	2434	0.73 (0.65–0.81)	0.70 (0.63–0.78)	2380	1693	0.84 (0.59–1.19)
Age 20–24 years	7,332,713	1310	0.81 (0.71–0.92)	0.76 (0.67–0.87)	1188	856	1.01 (0.64–1.62)
Age 25–30 years	5,491,130	843	1.04 (0.89–1.20)	0.96 (0.83–1.12)	619	477	1.22 (0.67–2.24)

All analyses were based on the Birth Cohort. Model 1. Adjusted for age (time axis), calendar year, sex. Model 2. Additionally adjusted for family history of diabetes, education (parents highest), maternal BMI (−25, 25–30, 30+). The sibling analyses were matched on age and adjusted for sex.

### Smoking during adulthood and type 1 diabetes

The HR of type 1 diabetes in smokers compared to non-smokers was 1.10 (CI 0.91–1.34) in the Military Conscription Cohort, 1.20 (CI 0.99–1.46) in the Pregnancy Cohort and 1.14 (CI 1.00–1.31) when the cohorts were combined, with no indication of a dose-response relationship (Table 2, Fig. 3). Adjustment for maternal smoking did not influence the results (Appendix Table S3). Snus use was not associated with type 1 diabetes (Table 2). In the sibling analyses (combined cohorts), the OR of type 1 diabetes was 1.59 (CI 1.08–2.35) in individuals who smoked compared to their non-smoking siblings (Table 2, Fig. 3). Performing the cohort analysis according to family history of diabetes indicated that smoking was associated with type 1 diabetes in those with (HR 1.34, CI 1.03–1.75) but not in those without diabetes in the family (HR 1.08, CI 0.93–1.27) (Fig. 3).

**Table 2:** Adult smoking, snus use and incidence of type 1 diabetes between age 19 and 30. Cohort and Sibling analyses.

	Person-Years/No. siblings	No. cases	HR/OR (95% CI) Model 1	HR/OR (95% CI) Model 2
**Cohort analysis-Military Conscription Cohort**				
Non-smoking	4,013,629	686	Ref	ref
Smoking	705,284	137	1.05 (0.92–1.20)	1.10 (0.91–1.34)
1–9 cigarettes/day	488,438	97	1.16 (0.94–1.44)	1.13 (0.91–1.41)
10–19 cigarettes/day	170,854	30	1.03 (0.71–1.48)	0.99 (0.68–1.44)
≥20 cigarettes/day	45,992	10	1.27 (0.68–2.38)	1.23 (0.66–2.31)
Non-snus user	3,517,693	601	ref	ref
Snus user	1,201,221	222	1.07 (0.92–1.25)	0.97 (0.82–1.13)
**Cohort analysis-Pregnancy Cohort**				
Non-smoking	4,318,239	292	ref	ref
Smoking	1,813,529	159	1.29 (1.06–1.57)	1.20 (0.99–1.46)
1–9 cigarettes/day	46,276	88	1.26 (0.99–1.60)	1.19 (0.93–1.51)
≥10 cigarettes/day	50,753	71	1.34 (1.03–1.73)	1.22 (0.94–1.59)
**Cohort analysis-Military Conscription and Pregnancy Cohorts combined**				
Non-smoking	8,318,064	975	ref	ref
Smoking	2,516,708	296	1.21 (1.06–1.38)	1.14 (1.00–1.31)
1–9 cigarettes/day	1,531,209	185	1.20 (1.03–1.41)	1.15 (0.98–1.35)
≥10 cigarettes/day	985,499	111	1.21 (0.99–1.48)	1.13 (0.92–1.38)
**Sibling analysis-Military Conscription and Pregnancy Cohorts combined**				
Non-smoking	393	304	ref	ref
Smoking	112	104	1.58 (1.07–2.32)	1.59 (1.08–2.35)
1–9 cigarettes/day	60	65	1.79 (1.13–2.82)	1.80 (1.14–2.84)
≥10 cigarettes/day	52	39	1.30 (0.76–2.22)	1.32 (0.77–2.26)

Results based on the Military Conscription and Pregnancy Cohorts. Model 2 is additionally adjusted for family history of diabetes, parents’ education, and adult BMI (only adult BMI in the sibling analysis). The Military Conscription Cohort is additionally adjusted for muscle strength, physical fitness (W_max_) and snus us.

### Sensitivity analyses

Further adjustment for early life factors and country of birth did not change any of the observed associations and the results also remained unchanged when we excluded everyone with missing data on co-variates and did complete case analysis with and without adjustment for covariates (Appendix Table S4). Combining information on adult and prenatal exposure to smoking revealed a reduced risk of adult-onset type 1 diabetes only in those exposed to maternal smoking who did not smoke themselves as adults (Appendix Table S3). Results based on the Military Conscription Cohort did not indicate that the combination of smoking and snus use conferred excess risk of type 1 diabetes (Appendix Table S3). Sensitivity analyses using a stricter definition of type 1 diabetes (never being prescribed an oral glucose lowering drug indicated similar results regarding associations with prenatal and adult exposure to smoking (Appendix Table S5).

## Discussion

In this large nationwide Swedish study, we found that individuals who were exposed to maternal smoking prenatally had a reduced risk of developing type 1 diabetes during childhood, whereas the association subsided in adults. Smoking as an adult did not appear to lower the risk; in fact, there was some evidence to suggest that it may even raise the risk of type 1 diabetes with adult-onset. These observations were supported by sibling analyses that could account for confounding from genetic and environmental factors shared within families.

This is the first study that investigates maternal smoking during pregnancy in relation to type 1 diabetes in the offspring beyond childhood. In line with previous reports based on the same data and other observational studies, there was a reduced risk of type 1 diabetes in children whose mothers smoked during pregnancy.^1^, ^2^, ^3^ The risk reduction we observed during childhood was in the order of 20–30% and evident until age 14 in both cohort and sibling analyses. The sibling analyses, which we regarded as having the highest evidentiary value, indicated that prenatal exposure to smoking is unrelated to the risk of developing type 1 diabetes as an adult. Furthermore, there was no indication that smoking as an adult confers protection against development of type 1 diabetes. Interestingly, the few studies that investigated incidence of type 1 diabetes in children in relation to parental smoking during childhood did not find an association.^1^^,^^2^ Taken together, this indicates that if smoking has the potential to reduce the risk of type 1 diabetes, such an effect is restricted to prenatal exposure and to type 1 diabetes that develops during childhood. Our findings also extend previous research by investigating potential effect modification by family history of diabetes and we find no indication that the reduced risk conferred by smoking is contingent on diabetes heritability.

There is no evidence from earlier studies that maternal smoking during pregnancy would lower the risk of some other immune mediated diseases, including rheumatoid arthritis, Multiple Sclerosis, Inflammatory Bowel Diseases, Systemic lupus erythematosus, or Psoriasis.^14^, ^15^, ^16^, ^17^, ^18^ Smoking during pregnancy is however associated with a reduced risk of celiac disease in the offspring.^19^ In this context it is noteworthy that there are similarities between celiac disease and type 1 diabetes as antibody positivity tends to occur during the first years of life in both conditions, genetic susceptibility is mainly conferred by overlapping variants in the human leukocyte antigen (HLA) complex, and they also share some environmental risk factors like breastfeeding practices and viral infections.^20^ Notably, maternal smoking during pregnancy is not associated with the risk of type 2 diabetes in the offspring.^21^ This suggests that the mechanisms involve pathways that are specific to autoimmune diabetes.

Experimental studies provide some support for beneficial effects of smoking on the development of autoimmune diabetes primarily relating to nicotine's immunosuppressive properties. It has been shown that nicotinic acetylcholine receptors (nAChRs) in T cells can be activated by nicotine and such activation can suppress systemic inflammation and autoimmunity, and such effects may persist postnatally.^5^^,^^22^, ^23^, ^24^ In type 1 diabetes-prone mouse models, nicotine-induced immunosuppression is linked to preserved insulin content and lower incidence of the disease.^6^ In childhood type 1 diabetes, the autoantibodies that marks the start of the autoimmune process tend to manifest within the first two years of life, lending credence to the theory that the etiology may include early life exposures.^25^ In this context it is noteworthy that most children with multiple autoantibodies progress to type 1 diabetes within 15 years.^26^ The autoimmune process leading to adult-onset type 1 diabetes may start later in life, thus being less affected by prenatal exposures. This could explain why the inverse association between prenatal exposure to smoking and type 1 diabetes did not last into adulthood.

Our findings suggest that smoking may increase the risk of adult-onset type 1 diabetes. We are only aware of one, very small, previous study on the topic and in contrast to us, they found a reduced risk in smokers.^7^ However, our findings align with previous observations regarding latent autoimmune diabetes in adults (LADA); pooled data from two Scandinavian Cohorts revealed a 30% higher risk of LADA in smokers and Mendelian Randomization analyses support that this reflects a causal effect.^27^ The mechanisms may involve direct adverse effects of smoking/nicotine exposure on insulin sensitivity.^28^ Insulin resistance has been implicated in the pathogenesis of type 1 diabetes primarily by increasing the demand on the beta-cells, which may accelerate the autoimmune process and progression to manifest diabetes.^29^ In support hereof, smoking was positively associated with insulin resistance in LADA.^27^ There was some indication that smoking may primarily promote type 1 diabetes in individuals with genetic susceptibility to diabetes as indicated by family history of diabetes. This finding also aligns with observations in LADA and warrants further exploration.^27^ Our study contributed to the limited number of studies that investigate potential modifiable risk factors for adult-onset type 1 diabetes. This is significant because, contrary to the widely held perception that type 1 diabetes primarily affects children, a recent study estimated that the median age at diagnosis is 29 years.^30^

This study provides a comprehensive picture of the influence of smoking exposure on type 1 diabetes by exploring smoking exposure in both early and later life in relation to type 1 diabetes diagnosed at different ages. We used nationwide registers and more than 3 million individuals for analyses of maternal smoking and 1.6 million individuals for adult smoking. To minimize loss to follow-up and ensure identification of all incident diabetes patients, we combined three high quality registers: the Patient, Prescribed Drug, and Diabetes Registers. Regarding the classification of diabetes type, it should be noted that a diagnosis of type 1 diabetes in the Diabetes Register is shown to be accurate in 97% of cases diagnosed before the age of 30.^11^ It should also be noted that anyone, ever recorded as having type 2 diabetes in any register, was not included as a case. Concerns about potential misclassification of type 2 diabetes did however preclude us from investigating type 1 diabetes with onset at higher ages. We had information on a range of potential confounders and could also adjust the analysis of adult smoking for maternal smoking and vice versa. Still, we did not have information on breastfeeding, exposure to infections or early dietary factors which may influence the risk of type 1 diabetes during childhood, and also lacked information on adult lifestyle factors besides BMI, smoking and physical fitness. The family-based design, where siblings that were discordant for diabetes and smoking exposure were compared, is a particular strength because it enables us to account for unmeasured environmental and genetic factors shared within families. This design is especially relevant when investigating early life exposures since this is the time siblings live together, while it may be less powerful in reducing confounding by adult lifestyle factors acquired while the siblings lived apart. It should be noted that we had less power in the sibling analyses, especially when investigating adult-onset type 1 diabetes. Limitations include the use of self-reported information on smoking, which may be especially problematic for smoking during pregnancy due to social desirability. The prospective design does however imply that potential misclassification is non-differential and as such, tends to dilute the associations. Another limitation is the lack of information on paternal smoking during pregnancy and parental smoking during childhood. To what extent the results are generalizable to populations outside of Scandinavia is not clear.

### Conclusion

Our findings indicate that prenatal exposure to smoking has the potential to reduce the risk of type 1 diabetes during childhood but not during adulthood. The clinical significance of this finding is difficult to see given the variety of major adverse health impacts smoking during pregnancy has on fetal and childhood health.^31^ Nevertheless, our findings contribute to the understanding of the etiology of type 1 diabetes and point at an etiologically important period, i.e., during fetal development. Our finding that smokers have an increased risk of adult-onset type 1 diabetes warrants further investigations.

## Contributors

SC, TA, and YW designed the study. TA and YW analyzed the data. SC drafted the manuscript. TA and YW directly accessed and verified the underlying data reported in the manuscript. All authors made substantial contributions to the interpretation of data, revising the manuscript for important intellectual content, and approved the final version to be published. All authors had full access to all the data in the study and accept responsibility to submit for publication.

## Data sharing statement

The data that support the findings of this study are available from Statistics Sweden and the Swedish National Board of Health and Welfare, but restrictions apply to the availability of these data, which were used under license for the current study, and so are not publicly available.

## Declaration of interests

None to declare.
