# Supplementary material for: Prenatal and adult exposure to smoking and incidence of type 1 diabetes in children and adults–a nationwide cohort study with a family-based design
Source: Lancet Reg Health Eur. 2023 Nov 4;36:100775. doi: 10.1016/j.lanepe.2023.100775 (PMC10652139; doi:10.1016/j.lanepe.2023.100775)
Supplement: Appendix Tables S1–S5 [file mmc1.docx]

## Appendix material for the manuscript “Prenatal and adult exposure to smoking and incidence of type 1 diabetes in children and adults-A nationwide study with a family-based design” by Wei et al.

1. **Appendix table 1. Characteristics of the three study populations.**
2. **Appendix table 2. Characteristics of the siblings with and without diabetes within the three study populations.**
3. **Appendix table 3. Prenatal and adult exposure to smoking, snus use and incidence of adult-onset type 1 diabetes. Mutual adjustments, and combined exposures.**
4. **Appendix table 4. Prenatal and adult exposure to smoking and incidence of type 1 diabetes. Complete case analyses and additional adjustment for early life factors and country of birth.**
5. **Appendix table 5. Prenatal and adult exposure to smoking and incidence of type 1 diabetes. Sensitivity analysis based on a stricter definition of type 1 diabetes (no prescription of oral glucose lowering drugs from 2005 onwards).**

**Appendix table 1. Characteristics of the three study populations.**

|  | **For analyses of prenatal exposure to smoking** | | | | **For analyses of adult exposure to smoking** | | | |  |
| --- | --- | --- | --- | --- | --- | --- | --- | --- | --- |
|  | **Birth cohort** | | | | **Military Conscription Cohort** | | **Pregnancy Cohort** | |  |
|  | Total cohort | | Type 1 diabetes | Total cohort | | Type 1 diabetes | Total cohort | Type 1 diabetes |  |
|  |  | |  |  | |  |  |  |  |
| Total number | 3 170 386 | | 18 745 | 406 593 | | 823 | 1 201 698 | 451 |  |
| Men No· (%) | 1 629 096 (51·4) | | 10 498 (56·0) | 394 705 (97·1) | | 806 (97·9) | ·· | ·· |  |
| Women, No· (%) | 1 541 290 (48·6) | | 8 247 (44·0) | 11 888 (2·9) | | 17 (2·1) | 1 201 698 | 451 |  |
| Birth years | 1982-2014 | | 1982-2014 | 1971-1992 | | 1978-1992 | 1951-2000 | 1953-1995 |  |
| Mean age (SD) | - | | - | 18·3 (0·6) | | 18·3 (0·4) | 25·7 (3·3) | 23·4 (3·1) |  |
|  | |  |  |  | |  |  |  |  |
| Family history of diabetes No· (%) | 56 836 (1·8) | | 1 681 (9·0) | 27 196 (6·7) | | 144 (17·5) | 91 992 (7·7) | 82 (18·2) |  |
|  |  | |  |  | |  |  |  |  |
| BMI Mean (SD)* | 23·9 (4·3) | | 24·1 (4·3) | 22·8 (3·5) | | 22·8 (3·7) | 23·3 (4·0) | 23·7 (4·3) |  |
| <25, No· (%) | 1 772 387 (69·7) | | 10 001 (68·0) | 252 967 (80·0) | | 500 (79·6) | 716 145 (75·0) | 247 (71·6) |  |
| 25·0-29·9, No· (%) | 547 227 (21·5) | | 3 299 (22·4) | 49 548 (15·7) | | 98 (15·6) | 174 506 (18·3) | 67 (19·4) |  |
| ≥30, No· (%) | 224 787 (8·8) | | 1 409 (9·6) | 13 814 (4·4) | | 30 (4·8) | 64 395 (6·7) | 31 (9·0) |  |
| Missing, No· (%) | 625 985 (19·7) | | 4 036 (21·5) | 90 264 (22·2) | | 195 (23·7) | 246 652 (20·5) | 106 (23·5) |  |
|  |  | |  |  | |  |  |  |  |
| Educational levelⱡ |  | |  |  | |  |  |  |  |
| Primary school, No· (%) | 123 986 (3·9) | | 617 (3·3) | 26 193 (6·5) | | 42 (5·2) | 208 517 (23·1) | 90 (24·3) |  |
| Secondary school, No· (%) | 1 309 956 (41·5) | | 8 435 (45·7) | 184 448 (45·9) | | 406 (49·9) | 450 993 (49·9) | 197 (53·1) |  |
| University, No· (%) | 1 723 783 (54·6) | | 9 420 (51·0) | 190 872 (47·5) | | 366 (45·0) | 245 021 (27·1) | 84 (22·6) |  |
| Missing, No· (%) | 12 661 (0·4) | | 273 (1·5) | 5 080 (1·2) | | 9 (1·1) | 297 167 (24·7) | 80 (17·7) |  |
|  |  | |  |  | |  |  |  |  |
| Muscle strength, Mean (SD) | ·· | | ·· | 633 (166) | | 632 (366) | ·· | ·· |  |
| Physical fitness (Wmax) Mean (SD) | ·· | | ·· | 267 (36) | | 267 (33) | ·· | ·· |  |
| % exposed to maternal smoking/smokers | 520 010 (10·5) | | 2 628 (9·2) | 60 765 (14·9) | | 137 (16·6) | 301 103 (25·1) | 159 (35·3) |  |
| % snus users | ·· | | ·· | 102 842 (25·3) | | 222 (27·0) | ·· | ·· |  |
|  |  | |  |  | |  |  |  |  |
| *Mother´s BMI for the Birth Cohort and own BMI for the Military Conscription and Pregnancy Cohorts. ⱡ Parent´s highest education. No (Number), SD (Standard deviation). | | | | | | | | | |

**Appendix table 2. Characteristics of the siblings with and without diabetes within the three study populations**

|  | **For analyses of prenatal exposure to smoking** | | **For analyses of adult exposure to smoking** | |
| --- | --- | --- | --- | --- |
|  | **Birth cohort** | | **Military Conscription and Pregnancy Cohort** | |
|  | All | Type 1 diabetes | All | Type 1 diabetes |
| Total number | 31 366 | 13 055 | 913 | 408 |
| Men No· (%) | 16 859 (53·7) | 7 337 (56·2) | 548 (60·0) | 270 (66·2) |
| Women, No· (%) | 14 507 (46·3) | 5 718 (43·8) | 365 (40·0) | 138 (33·8) |
| Age Mean (SD) | 10·9 (6·3) | 11·0 (6·4) | 26·1 (3·5) | 26·0 (3·5) |
| Birth years | 1982-2014 | 1982-2014 | 1953-1995 | 1953-1992 |
|  |  |  |  |  |
| Family history of diabetes No· (%) | 11 259 (35·9) | 1 951 (14·9) | 418 (45·8) | 102 (25·0) |
|  |  |  |  |  |
| BMI Mean (SD)* | 24·0 (4·3) | 24·1 (4·3) | 22·7 (3·8) | 22·6 (3·8) |
| <25, No· (%) | 16 503 (68·2) | 6 864 (67·4) | 566 (75·9) | 244 (75·1) |
| 25·0-29·9, No· (%) | 5 458 (22·5) | 2 344 (23·0) | 127 (17·0) | 60 (18·5) |
| ≥30, No· (%) | 2 250 (9·3) | 970 (9·5) | 53 (7·1) | 21 (6·5) |
| Missing, No· (%) | 7 155 (22·8) | 2 877 (22·0) | 167 (18·3) | 83 (20·3) |
|  |  |  |  |  |
| Educational levelⱡ |  |  |  |  |
| Primary school, No· (%) | 873 (2·8) | 325 (2·5) | 124 (14·1) | 46 (11·6) |
| Secondary school, No· (%) | 14 147 (45·7) | 5 789 (44·9) | 443 (50·2) | 203 (51·4) |
| University, No· (%) | 15 958 (51·5) | 6 788 (52·6) | 315 (35·7) | 146 (37·0) |
| Missing, No· (%) | 388 (1·2) | 153 (1·2) | 31 (3·4) | 13 (3·2) |
|  |  |  |  |  |
| % exposed to maternal smoking/smoker | 3 765 (12·0) | 1 483 (11·4) | 216 (23·7) | 104 (25·5) |

*Mother´s BMI for the Birth Cohort and own BMI for the Military Conscription and Pregnancy Cohorts. ⱡ Parent´s highest education. No (Number), SD (Standard deviation).

Appendix table 3. **Prenatal and adult exposure to smoking, snus use and incidence of adult-onset type 1 diabetes. Mutual adjustments, and combined exposures.**

|  | **Cohort analyses** | | |  | **Sibling analysis** | | |
| --- | --- | --- | --- | --- | --- | --- | --- |
|  | **Person-years** | **No. cases** | **HR (95% CI)**  **Model 1** | **HR (95% CI)**  **Model 2** | **No. siblings** | **No. cases** | **OR (95% CI)** |
| **Prenatal exposure to smoking adjusted for adult smoking (yes vs.no)** | | | | | | | |
| Maternal non-smoking | 2 818 717 | 477 | ref | ref | 143 | 123 | ref |
| Maternal smoking | 1 169 650 | 176 | 0·92 (0·78-1·10) | 0·92 (0·78-1·07) | 50 | 47 | 1·52 (0·45-5·08) |
| 1-9 cigarettes/day | 715 356 | 100 | 0·85 (0·69-1·06) | 0·82 (0·66-1·02) | 26 | 23 | 1·46 (0·42-5·07) |
| ≥10 cigarettes/day | 454 293 | 76 | 1·04 (0·81-1·32) | 0·96 (0·75-1·23) | 24 | 24 | 1·67 (0·41-6·85) |
|  |  |  |  |  |  |  |  |
| **Adult exposure to smoking adjusted for prenatal exposure to smoking (yes vs. no)** | | | | | | | |
| Non-smoking | 3 248 388 | 527 | ref | ref |  |  |  |
| Smoking | 739 979 | 126 | 1·20 (0·98-1·46) | 1·10 (0·92-1·33) |  |  |  |
| 1-9 cigarettes/day | 437 453 | 79 | 1·20 (0·95-1·52) | 1·17 (0·92-1·49) |  |  |  |
| ≥10 cigarettes/day | 302 525 | 47 | 1·19 (0·88-1·62) | 1·16 (0·84-1·58) |  |  |  |
|  |  |  |  |  |  |  |  |
| **Prenatal and adult exposure to smoking (yes vs. no)** | | | | |  |  |  |
| Maternal non-smoking/adult non-smoking | 2 412 737 | 416 | ref | ref |  |  |  |
| Maternal smoking/adult non-smoking | 835 652 | 111 | 0·78 (0·64-0·97) | 0·76 (0·62-0·94) |  |  |  |
| Maternal non-smoking/adult smoking | 405 981 | 61 | 0·98 (0·74-1·28) | 0·93 (0·71-1·22) |  |  |  |
| Maternal smoking/adult smoking | 333 998 | 65 | 1·33 (1·02-1·74) | 1·24 (0·94-1·62) |  |  |  |
|  |  |  |  |  |  |  |  |
| **Adult exposure to smoking and snus (yes vs. no)** | | | | |  |  |  |
|  | Snus use |  |  |  |  |  |  |
| Non-smoking/no snus use | 3 014 981 | 513 | ref | ref |  |  |  |
| Smoking/no snus use | 381 835 | 72 | 1·11 (0·87-1·43) | 1·05 (0·82-1·36) |  |  |  |
| Non-smoking/snus use | 884 625 | 157 | 1·04 (0·87-1·25) | 1·03 (0·86-1·23) |  |  |  |
| Smoking/snus use | 309 670 | 64 | 1·21 (0·94-1·58) | 1·15 (0·88-1·50) |  |  |  |
|  |  |  |  |  |  |  |  |

Model 1 is Adjusted for age (time axis), calendar year, sex. Model 2 for analyses of prenatal exposure to smoking is additionally adjusted for family history of diabetes, parents´ education, adult BMI, and adult smoking. Model 2 for analyses of adult exposure to smoking was additionally adjusted for family history of diabetes, parents´ education, adult BMI, maternal smoking, and maternal BMI. Model 2 for analyses of combined exposure to smoking and snus use were additionally adjusted for family history of diabetes, parents’ education, and adult BMI, muscle strength and physical fitness (Wmax). The sibling analyses were matched on age and adjusted for sex, adult BMI and adult smoking. The numbers did not allow us to perform sibling analyses of adult exposure to smoking in this subset. Analyses of the combination of prenatal and adult smoking were based on members of the Pregnancy Cohort or Military Conscription Cohort born 1983-2000 who were also members of the Birth cohort. Analyses of combined use of snus and smoking were based on members of the Military Conscription Cohort.

**Appendix table 4. Prenatal and adult exposure to smoking and incidence of type 1 diabetes. Complete case analyses and additional adjustment for early life factors and country of birth (cohort analysis).**

|  |  | Person-years | No· cases | HR (95% CI)  Model 1 | HR (95% CI)  Model 2 |
| --- | --- | --- | --- | --- | --- |
| **PRENATAL EXPOSURE TO SMOKING** | | | | | |
| **Type 1 diabetes during childhood (age 0 – 18)** | | | | | |
| Complete case analyses | Maternal Non-smoking | 30 550 799 | 11 363 | ref | ref |
|  | Maternal smoking | 5 825 680 | 1 510 | 0·76 (0·72-0·80) | 0·73 (0·69-0·77) |
| Additional adjustment for early life factors* and maternal country of birth | Maternal Non-smoking | 39 515 944 | 13 834 |  |  |
|  | Maternal smoking | 8 809 202 | 1 998 | 0·73 (0·69-0·76) | 0·72 (0·68-0·76) |
|  | | | | | |
| **Type 1 diabetes during adulthood (age 19 – 30)** | | | | | |
| Complete case analyses | Maternal Non-smoking | 7 009 590 | 1 260 | ref | ref |
|  | Maternal smoking | 2 368 576 | 368 | 0·90 (0·80-1·01) | 0·88 (0·78-0·99) |
| Additional adjustment for early life factors* and maternal country of birth | Maternal Non-smoking | 10 550 350 | 1 909 |  |  |
|  | Maternal smoking | 3 604 050 | 563 | 0·89 (0·81-0·98) | 0·84 (0·77-0·93) |
|  | | | | | |
| **ADULT EXPOSURE TO SMOKING** | | | | | |
| **Type 1 diabetes during adulthood (age 19-30)** | | | | | |
| Complete case analyses | Non-smoking | 5 542 186 | 727 | ref | ref |
|  | Smoking | 1 443 868 | 180 | 1·16 (0·98-1·37) | 1·12 (0·87-1·45) |
| Additional adjustment for country of birth | Non-smoking | 8 318 064 | 975 |  |  |
|  | Smoking | 2 516 708 | 296 | 1·21 (1·06-1·38) | 1·15 (1·00-1·31) |
| Analyses of prenatal exposure were based on the Birth Cohort and analyses of adult exposure were based on combined data from the Military Conscription and Pregnancy Cohorts. Model 1. Adjusted for age (time axis), calendar year, sex. Model 2. Additionally adjusted for family history of diabetes, parents´ education, maternal BMI/own BMI. *maternal age at delivery, birth order, gestational age and birth weight for gestational age. | | | | | |

**Appendix table 5. Prenatal and adult exposure to smoking and incidence of type 1 diabetes. Sensitivity analysis based on a stricter definition of type 1 diabetes (no prescription of oral glucose lowering drugs from 2005 onwards).**

|  | Person-years | No. cases | HR/OR (95% CI) |
| --- | --- | --- | --- |
| **MATERNAL SMOKING VS NON-SMOKING AND TYPE 1 DIABETES BY AGE** | | | |
| Age 0-4 years | 15 794 419 | 3 308 | 0·74 (0·66-0·83) |
| Age 5-9 years | 14 437 819 | 5 291 | 0·69 (0·64-0·76) |
| Age 10-14 years | 11 792 969 | 5 191 | 0·72 (0·66-0·78) |
| Age 15-19 years | 9 440 519 | 2 424 | 0·69 (0·62-0·77) |
| Age 20-24 years | 7 332 713 | 1 266 | 0·75 (0·66-0·86) |
| Age 25-30 years | 5 491 130 | 788 | 0·93 (0·80-1·09) |
|  |  |  |  |
| **ADULT EXPOSURE TO SMOKING** | | | |
| Non-smoking | 8 318 064 | 899 | ref |
| Smoking | 2 516 708 | 251 | 1·09 (0·94-1·25) |
| 1-9 cigarettes/day | 1 531 209 | 157 | 1·08 (0·91-1·29) |
| ≥10 cigarettes/day | 985 499 | 94 | 1·09 (0·87-1·35) |
| ***Individuals with family history of diabetes*** | | | |
| Non-smoking | 863 114 | 190 | ref |
| Smoking | 308 645 | 66 | 1·28 (0·95-1·71) |
| ***Sibling analysis*** | **No. Siblings** | **No. Cases** |  |
| Non-smoking | 345 | 278 | ref |
| Smoking | 93 | 81 | 1·40 (0·91-2·14) |
| 1-9 cigarettes/day | 46 | 50 | 1·71 (1·03-2·84) |
| ≥10 cigarettes/day | 47 | 31 | 1·04 (0·58-1·87) |
|  |  |  |  |

The analysis of prenatal exposure to smoking was based on the Birth Cohort and the HRs were adjusted for age (time axis), calendar year, sex, family history of diabetes, education, and maternal BMI. The analysis of adult smoking was based on combined data from the Military Conscription and Pregnancy Cohorts and the HRs were adjusted for age (time axis), calendar year, sex, family history of diabetes, parents’ education, and BMI. The sibling analysis were matched on age and adjusted for sex (and BMI in the analysis of adult exposure to smoking).
